# Supplementary material for: Early Life Short-Term Exposure to Polychlorinated Biphenyl 126 in Mice Leads to Metabolic Dysfunction and Microbiota Changes in Adulthood
Source: Int J Mol Sci. 2022 Jul 26;23(15):8220. doi: 10.3390/ijms23158220 (PMC9330872; doi:10.3390/ijms23158220)
Supplement: Supplementary file 1 [file ijms-23-08220-s001.zip › ijms-1829056-supplementary.pdf]

Early-life exposure to polychlorinated biphenyl 126 in mice leads to metabolic dysfunction and microbiota changes in adulthood

Yuan Tian[1], Bipin Rimal[1], Wei Gui[2], Imhoi Koo[1], Shigetoshi Yokoyama[1], Gray H. Perdew[1], Andrew D. Patterson[1,*]

[1]Department of Veterinary and Biomedical Sciences, The Pennsylvania State University, University Park, PA 16802, United States

[2]Huck Institutes of the Life Sciences, The Pennsylvania State University, University Park, PA 16802, United States

[*]To whom correspondence should be addressed. Email: adp117@psu.edu. Address: 322 Life Science Bldg, University Park, PA 16802, Phone: 814-867-4565

Table S1. Serum cytokine (32-Plex) assay results.

Unit: pg/mL	6th day				13th week		
	Vehicle	PCB 126	P value		Vehicle	PCB 126	P value
**Eotaxin**	13646 ± 3784	12163 ± 1254	0.40		13794 ± 3276	15966 ± 3008	0.26
**G-CSF**	326.5 ± 84.8	327.3 ± 53.3	0.98		512.3 ± 192.5	618.8 ± 186.5	0.35
**GM-CSF**	22.7 ± 4.0	23.7 ± 5.6	0.73		25.2 ± 6.1	23.5 ± 2.1	0.55
**IFNγ**	34.7 ± 7.1	36.0 ± 4.0	0.70		34.2 ± 5.3	33.2 ± 5.2	0.75
**IL-1α**	43.8 ± 15.4	43.7 ± 17.3	0.99		61.7 ± 50.4	32.3 ± 5.3	0.21
**IL-1β**	25.5 ± 19.3	20.5 ± 5.4	0.56		18.5 ± 4.1	18.3 ± 2.0	0.93
**IL-2**	23.0 ± 3.7	20.3 ± 4.7	0.30		20.3 ± 3.6	20.5 ± 4.3	0.94
**IL-3**	25.5 ± 3.8	28.5 ± 3.9	0.21		34.2 ± 11.9	31.3 ± 9.5	0.66
**IL-4**	33.4 ± 2.2	34.2 ± 7.5	0.82		37.7 ± 6.9	32.8 ± 5.0	0.20
**IL-5**	93.5 ± 51.7	49.5 ± 8.3	0.09		94.7 ± 59.1	70.8 ± 11.3	0.37
**IL-6**	47.2 ± 15.7	39.3 ± 3.9	0.28		62.7 ± 47.0	55.8 ± 25.7	0.76
**IL-7**	43.8 ± 4.5	41.0 ± 3.0	0.27		48.8 ± 11.5	40.8 ± 1.6	0.15
**IL-9**	40.5 ± 3.6	41.5 ± 10.0	0.83		50.0 ± 13.0	39.0 ± 4.8	0.10
**IL-10**	37.8 ± 14.9	33.0 ± 5.3	0.48		33.0 ± 4.7	34.8 ± 2.9	0.44
**IL-12p40**	31.8 ± 4.4	33.7 ± 3.4	0.44		35.7 ± 7.3	34.7 ± 13.8	0.88
**IL-12p70**	29.8 ± 3.5	25.8 ± 3.4	0.09		29.5 ± 4.1	25.3 ± 4.1	0.11
**IL-13**	42.6 ± 5.5	40.8 ± 3.9	0.56		41.3 ± 8.5	37.3 ± 3.9	0.33
**IL-15**	44.2 ± 3.0	42.3 ± 5.8	0.51		48.3 ± 11.9	43.2 ± 8.7	0.41
**IL-17**	48.5 ± 19.1	38.8 ± 5.4	0.28		43.0 ± 1.3	42.2 ± 10.8	0.86
**IP-10**	2745.7 ± 344.7	2469.8 ± 207.7	0.13		2669.3 ± 963.7	3075.2 ± 501.9	0.39
**KC**	125.2 ± 43.5	158.5 ± 32.4	0.17		114.3 ± 49.1	94.0 ± 21.9	0.39
**LIF**	40.4 ± 6.9	43.5 ± 4.3	0.41		40.3 ± 4.0	39.5 ± 2.6	0.68
**LIX**	4829.8 ± 2785.0	4763.7 ± 2037.8	0.96		3516.0 ± 4883.2	2371.3 ± 1466.6	0.60

**MCP-1**	22.0 ± 8.0	21.8 ± 3.3	0.96		24.2 ± 3.0	20.0 ± 4.6	0.10
**M-CSF**	31.2 ± 2.2	29.2 ± 3.7	0.29		33.5 ± 16.1	33.3 ± 6.8	0.98
**MIG**	733.6 ± 131.6	688.0 ± 218.1	0.68		627.0 ± 271.9	618.5 ± 174.9	0.95
**MIP-1α**	30.5 ± 5.4	34.7 ± 3.0	0.14		33.3 ± 7.0	33.8 ± 3.6	0.88
**MIP-1B**	59.3 ± 8.9	69.8 ± 9.6	0.08		68.3 ± 7.1	62.0 ± 14.0	0.35
**MIP-2**	40.0 ± 5.3	45.3 ± 4.0	0.08		44.0 ± 9.9	42.2 ± 5.6	0.70
**RANTES**	36.6 ± 10.3	50.5 ± 8.9	0.05		57.7 ± 21.7	50.5 ± 8.3	0.48
**TNFα**	28.3 ± 4.4	23.3 ± 3.5	0.06		25.3 ± 2.7	25.7 ± 4.3	0.87
**VEGF**	37.0 ± 8.4	42.2 ± 6.1	0.25		36.2 ± 7.2	40.7 ± 5.2	0.24

G-CSF: granulocyte colony stimulating factor; GM-CSF: Granulocyte/macrophage colony stimulating factor; IFNγ: interferon gamma; IL: interleukin; IP-10: interferon gamma inducible protein 10; KC: keratinocyte chemoattractant; LIF: leukaemia inhibitory factor; LIX: lipopolysaccharide-induced CXC chemokine; MCP-1: monocyte chemoattractant protein-1; M-CSF: macrophage colony-stimulating factor; MIG: monokine induced by IFN-γ; MIP: macrophage inflammatory protein; RANTES: regulated upon activation, normal T cell expressed and presumably secreted; TNFα: tumor necrosis factor alpha; VEGF: vascular endothelial growth factor.

Table S2. mRNA gene-targeted primers used in this study

Gene	Abbreviation	Sequence (5'-3')
Cytochrome P450, family 1, member A1	Cyp1a1	CTCTTCCCTGGATGCCTTGAA<br>GGATGTGGCCCTTCTCAAATG
Cytochrome P450, family 1, member A2	Cyp1a2	GCCCCTGCCCTTCAGTGGTACAG<br>AGGAGTGGAGCCGATGCGGA
Stearoyl-CoA desaturase-1	Scd1	TTCTTGCGATACACTCTGGTGC<br>CGGGATTGAATGTTCTTGTCGT
Cluster of differentiation 36	Cd36	TGGCCTTACTTGGGATTGG<br>CCAGTGTATATGTAGGCTCATCCA
Acetyl-CoA carboxylase alpha	Acaca	TAACAGAATCGACACTGGCTGGCT<br>ATGCTGTTCCTCAGGCTCACATCT
Carnitine palmitoyltransferase 1A	Cpt1a	CGTGACGTTGGAATC<br>TCTGCGTTTATGCCTATC
Diacylglycerol O-Acyltransferase 2	Dgat2	CGCAGCGAAAACAAGAATAA<br>GAAGATGTCTTGGAGGGCTG
Fatty acid synthase	Fasn	GGTGTGGTGGGTTTGGTGAATTGT<br>TCACGAGGTCATGCTTTAGCACCT
Cytochrome P450, family 7, subfamily A, polypeptide 1 (Cholesterol 7α-hydroxylase)	Cyp7a1	AGCAACTAAACAACCTGCCAGT<br>ACTAGTCCGGATATTCAAGGATGCA
Cytochrome P450, family 7, subfamily B, polypeptide 1 (Oxysterol 7α-hydroxylase)	Cyp7b1	TAGCCCTCTTTCCTCCACTCATA<br>GAACCGATCGAACCTAAATTCCT
Cytochrome P450, family 8, subfamily B, polypeptide 1 (Sterol 12α-hydroxylase)	Cyp8b1	GGCTGGCTTCCTGAGCTTATT<br>ACTTCCTGAACAGCTCATCGG
Cytochrome P450, family 27, subfamily A, polypeptide 1 (Sterol 27-hydroxylase)	Cyp27a1	GCCTCACCTATGGGATCTTCA<br>TCAAAGCCTGACGCAGATG
Aldo-keto reductase family 1 member D1	Akr1d1	TGCACACCACCAAATATCCCT<br>CTTCACTGCCACATAGGTCTTC
Cysteine dioxygenase	Cdo	GGGGACGAAGTCAACGTGG<br>ACCCCAGCACAGAATCATCAG
Cysteine sulfinate decarboxylase	Csad	CCAGGACGTGTTTGGGATTGT<br>ACCAGTCTTGACACTGTAGTGA
Taurine transporter	Taut	GCACACGGCCTGAAGATGA<br>ATTTTTGTAGCAGAGGTACGGG
Bile acid-CoA: amino acid N-acyltransferase	Baat	GGAAACCTGTTAGTTCTCAGGC<br>GTGGACCCCCATATAGTCTCC
Bile acid-CoA synthetase	Bacs	ACCCTGGATCAGCTCCTGGAT<br>GTTCTCAGCTAGCAGCTTGG
Hepatic nuclear factor 4α1	Hnf4a1	AAATGTGCAGGTGTTGACCA<br>CACGCTCCTCCTGAAGAATC
Phosphoenolpyruvate carboxykinase	Pepck	GGCCACAGCTGCTGCAG<br>GGTCGCATGGCAAAGGG
Glyceraldehyde-3-phosphate dehydrogenase	Gapdh	CCTCGTCCCGTAGACAAAATG

		TGAAGGGGTCGTTGATGGC

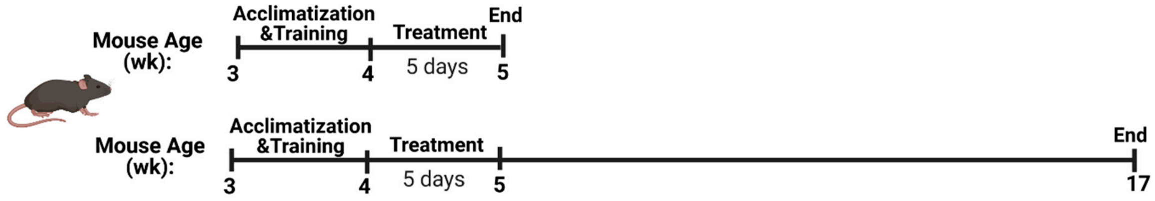

Figure S1. Experimental schedule of the present study.

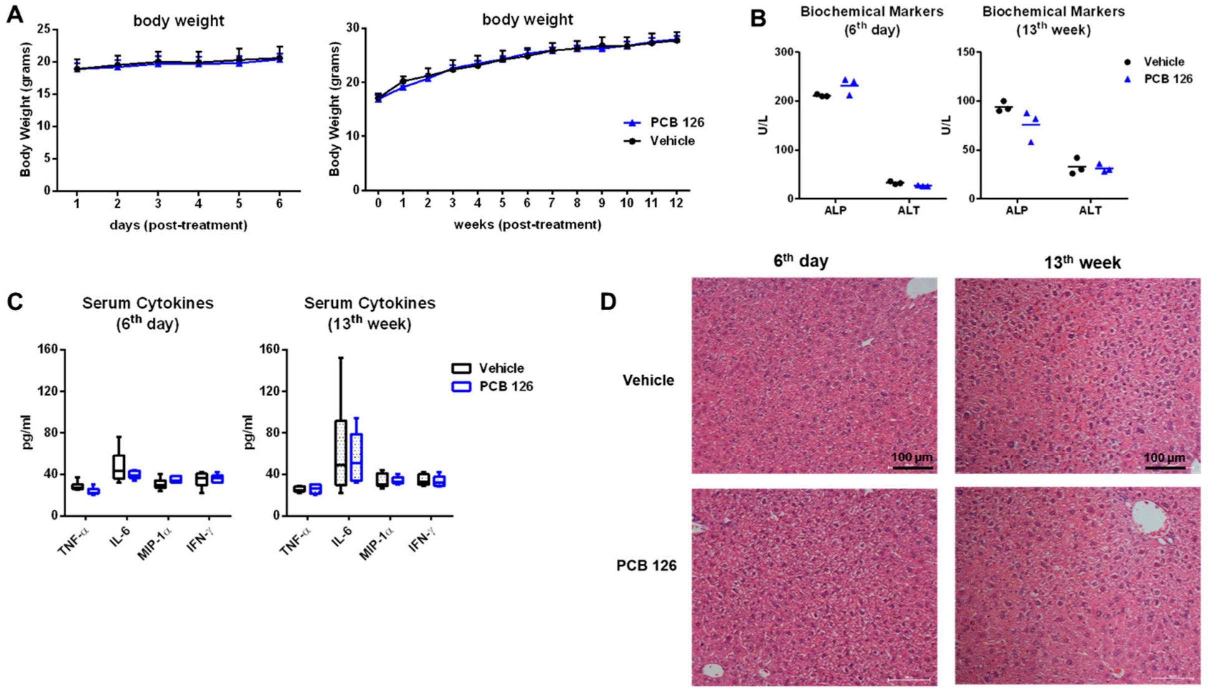

Figure S2. (**A**) Body weight of mice recorded from mice with vehicle or PCB 126 exposure. (**B**) Serum concentrations of alanine transaminase (ALT) and alkaline phosphatase (ALP) from mice with vehicle or PCB 126 exposure. (**C**) Serum cytokines from mice with vehicle or PCB 126 exposure. (**D**) Light microscopic examination of H&E-stained liver sections from mice with vehicle or PCB 126 exposure. Values are means ± S.D. or median and interquartile range (n = 3 per group, serum biochemical markers; n = 6 per group, other analysis).

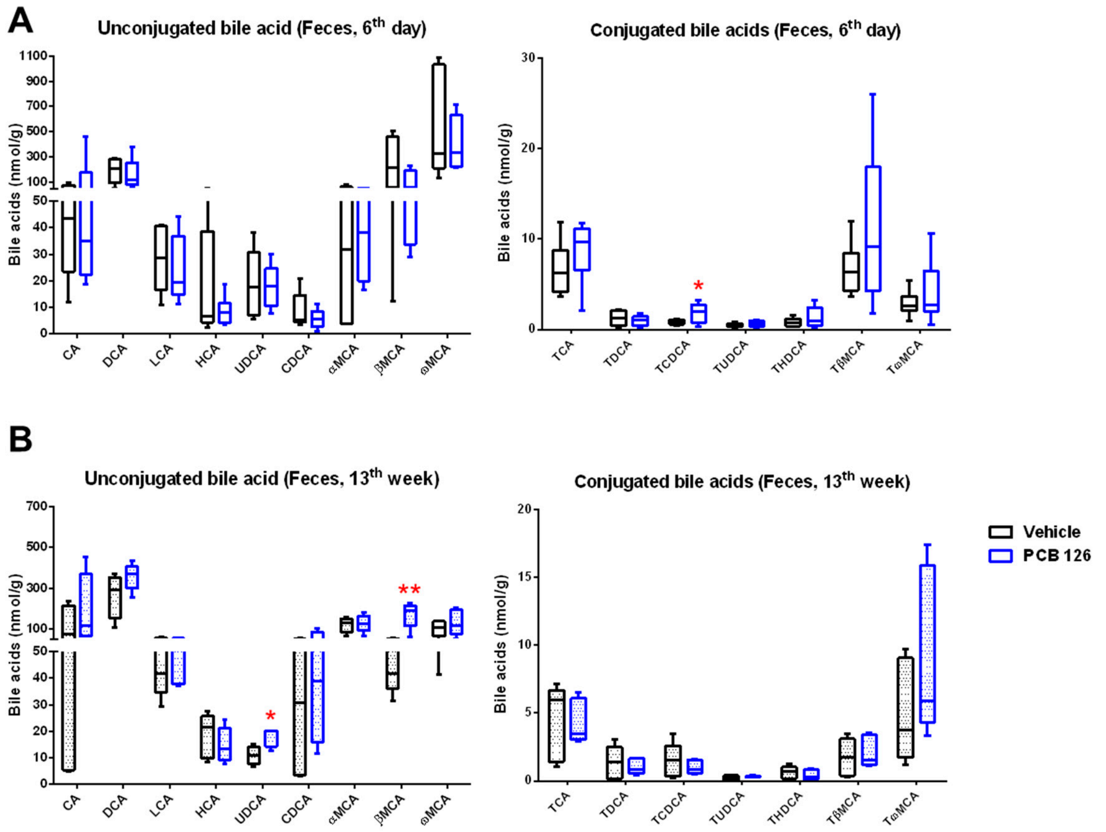

Figure S3. (**A**,**B**) Quantitative UHPLC-MS/MS analysis of bile acids in the feces from mice with vehicle or PCB 126 exposure. Values are median and interquartile range (n = 6 per group). * $P < 0.05$, ** $P < 0.01$ compared to vehicle.

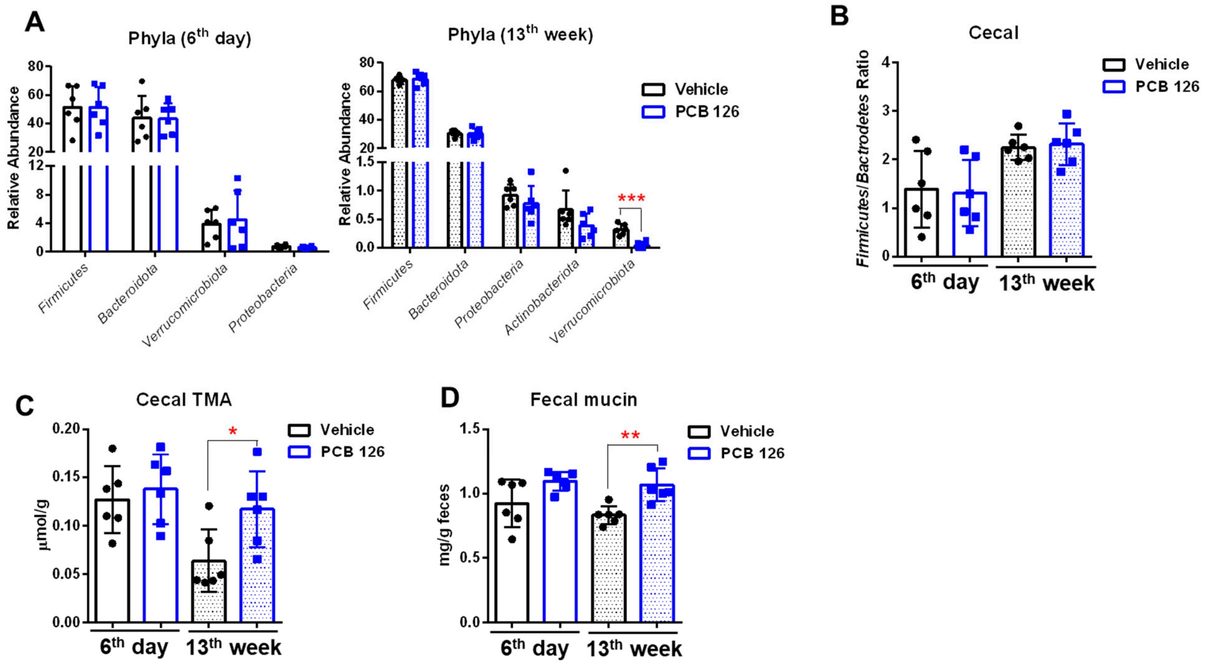

Figure S4. (**A**,**B**) Relative abundance of cecal bacteria from phylum (**A**) and ratio of *Firmicutes* to *Bacteroidetes* (**B**) in cecal content from mice with vehicle or PCB 126 exposure. (**C**) NMR analysis of cecal trimethylamine (TMA) from mice with vehicle or PCB 126 exposure. (**D**) Fecal mucin levels from mice with vehicle or PCB 126 exposure. Values are means ± S.D. (n = 6 per group). * $P < 0.05$, ** $P < 0.01$, *** $P < 0.001$ compared to vehicle.
